# Cardiovascular Complications following Chronic Treatment with Cocaine and Testosterone in Adolescent Rats

**DOI:** 10.1371/journal.pone.0105172

**Published:** 2014-08-14

**Authors:** Sheila A. Engi, Fábio C. Cruz, Rodrigo M. Leão, Luís C. Spolidorio, Cleopatra S. Planeta, Carlos C. Crestani

**Affiliations:** 1 Laboratory of Pharmacology, Department of Natural Active Principles and Toxicology, School of Pharmaceutical Sciences, Univ. Estadual Paulista-UNESP, Araraquara, SP, Brazil; 2 Joint UFSCar-UNESP Graduate Program in Physiological Sciences, São Carlos, SP, Brazil; 3 Behavioral Neuroscience Branch, Intramural Research Program, National Institute on Drug Abuse, US National Institutes of Health, Department of Health and Human Services, Baltimore, Maryland, United States of America; 4 Department of Physiology and Pathology, School of Dentistry of Araraquara, Univ. Estadual Paulista-UNESP, Araraquara, SP, Brazil; Vanderbilt University Medical Center, United States of America

## Abstract

Concomitant use of anabolic androgenic steroids and cocaine has increased in the last years. However, the effects of chronic exposure to these substances during adolescence on cardiovascular function are unknown. Here, we investigated the effects of treatment for 10 consecutive days with testosterone and cocaine alone or in combination on basal cardiovascular parameters, baroreflex activity, hemodynamic responses to vasoactive agents, and cardiac morphology in adolescent rats. Administration of testosterone alone increased arterial pressure, reduced heart rate (HR), and exacerbated the tachycardiac baroreflex response. Cocaine-treated animals showed resting bradycardia without changes in arterial pressure and baroreflex activity. Combined treatment with testosterone and cocaine did not affect baseline arterial pressure and HR, but reduced baroreflex-mediated tachycardia. None of the treatments affected arterial pressure response to either vasoconstrictor or vasodilator agents. Also, heart to body ratio and left and right ventricular wall thickness were not modified by drug treatments. However, histological analysis of left ventricular sections of animals subjected to treatment with testosterone and cocaine alone and combined showed a greater spacing between cardiac muscle fibers, dilated blood vessels, and fibrosis. These data show important cardiovascular changes following treatment with testosterone in adolescent rats. However, the results suggest that exposure to cocaine alone or combined with testosterone during adolescence minimally affect cardiovascular function.

## Introduction

Adolescence is a critical period of vulnerability for development of drug addiction [1]. Indeed, most experimental use of drugs begins during adolescence [2], [3]. Furthermore, epidemiological studies indicate that earlier onset of substance use predicts greater likelihood of development of substance use disorders [4]–[6]. These latter findings are associated with clinical and preclinical results demonstrating that adolescents are undergoing developmental changes in primary motivational circuitry, which can influence the amount of drug intake, psychopharmacological responsivity and the susceptibility to development of drug dependence [1], [7].

Adolescent vulnerability to drug abuse and addiction have been associated with several substances, including cocaine and androgenic–anabolic steroids (AAS) [5], [8]. Emerging data indicate that AAS use among adolescents is frequently associated with abuse of other substances, and cocaine is the drug most frequently coabused with AAS [8], [9]. These pieces of evidence are alarming because although numerous clinical and experimental reports document the adverse effects of cocaine and AAS abuse in adulthood, a limited number of studies have assessed the potential toxic effects of the abuse of these substances, specially their concomitant use, in adolescents.

The use of cocaine and AAS has been associated with severe medical consequences, including cardiovascular complications [10]–[12]. Cocaine’s acute effects on cardiovascular function are well described and include hypertension, coronary vasoconstriction and cardiac arrhythmias [10], [13]. Cardiovascular toxicity following chronic cocaine exposure is less understood, but clinical and preclinical studies have reported myocarditis, arrhythmias, alterations in baroreflex activity, and vascular dysfunctions [10], [14]. Studies in humans and animals have also associated chronic AAS administration with cardiovascular complications, including hypertension, atherosclerosis, cardiac pathologies, impairment of baroreflex activity and changes in vascular function [12], [14]–[16]. Interestingly, preclinical studies have demonstrated that concomitant AAS and cocaine treatment potentiate the cardiovascular effects of each other [17]–[19]. Indeed, we recently reported that administration of either testosterone or cocaine for 10 consecutive days in adult rats caused a range of cardiovascular effects (e.g., mild hypertension, arrhythmia, baroreflex changes, and alterations in stress-evoked autonomic responses), which were more pronounced when those substances were administrated concomitantly [14], [20].

Taken together, this evidence clearly demonstrates the occurrence of cardiovascular diseases following exposure to cocaine and AAS alone and combined. However, the influence of chronic exposure to these substances during adolescence on cardiovascular function has never been investigated. Adolescence has been defined in rodents as the ontogenic period from postnatal day 28 to 42 [21], although some authors have extended this period from the postnatal day 21 to 59 [22]. During this developmental period, the animals present adolescent-typical neurobehavioral characteristics [21], [23]. Therefore, our purpose in the present study was to investigate the effects of daily administration of testosterone and cocaine alone or in combination for 10 consecutive days on basal cardiovascular parameters, baroreflex activity, hemodynamic responses to vasoactive agents, and cardiac morphology in adolescent rats (28 days old).

## Materials and Methods

### Ethical approval and animals

Housing conditions and experimental procedures were carried out according to protocols approved by the Ethical Committee for Use of Animal of the School of Pharmaceutical Science/UNESP (permit number: 10/2011), which complies with the Brazilian and international guidelines for animal use and welfare. Twenty-eight day old male Wistar rats obtained from the animal breeding facility of the São Paulo State University-UNESP (Botucatu, SP, Brazil) were housed in plastic cages in a temperature-controlled room at 24°C in the Animal Facility of the Laboratory of Pharmacology, School of Pharmaceutical Sciences, São Paulo State University-UNESP. They were kept under a 12∶12 h light-dark cycle (lights on between 6∶00 am and 6∶00 pm) with free access to water and standard laboratory food.

### General experimental procedures

Animals were randomly divided into four groups: (i) vehicle (almond oil, 1 ml/kg, s.c.) + vehicle (0.9% NaCl, 1 ml/kg, i.p.); (ii) testosterone (10 mg/kg, s.c.) + vehicle; (iii) vehicle + cocaine (20 mg/kg, i.p.); and (iv) testosterone + cocaine. Animals received treatments once daily for 10 consecutive days (treatment from postnatal day 28 to 37). The doses and treatment regimen were chosen based on our previous studies [14], [24].

Forty-eight hours after the end of treatments, and 24 hours before the trial, rats were subjected to surgical preparation for evaluation of cardiovascular function. In the trial day, animals were transferred to the experimental room in their home box. They were allowed 60 min to adapt to experimental room conditions such as sound and illumination before starting cardiovascular recording. The experimental room was temperature controlled (24°C) and was acoustically isolated from the other rooms. Cardiovascular parameters were measured in awake, freely moving rats.

Animals in all experimental groups were subjected to a 30 min period of basal cardiovascular recording. In the sequence, they received intravenous infusion of phenylephrine and sodium nitroprusside. Infusions of vasoactive drugs were randomized and the second treatment was not begun before cardiovascular parameters returned to control values (interval between infusions was approximately 5 min). At the end of the experiments, animals were sacrificed and their hearts were removed, weighed and fixed for morphological analysis.

### Surgical Preparation

Rats were anesthetized with tribromoethanol (250 mg/kg, i.p.) and a catheter (a 4 cm segment of PE-10 heat-bound to a 10 cm segment of PE-50) (Clay Adams, Parsippany, NJ, USA) was inserted into the abdominal aorta through the femoral artery for cardiovascular recording. A second catheter was implanted into the femoral vein for the infusion of vasoactive agents to evoke arterial pressure changes. Both catheters were tunneled under the skin and exteriorized on the animal’s dorsum. After the surgery, rats were treated with a streptomycin and penicillin polyantibiotic formulation (0.27 mg/kg, i.m.; Pentabiotico, Fort Dodge, Campinas, SP, Brazil) to prevent infection, and received the non-steroidal anti-inflammatory drug flunixine meglumine (0.025 mg/kg, i.m.; Banamine, Schering-Plough, Cotia, SP, Brazil) for postoperative analgesia.

### Measurement of Cardiovascular Parameters

The arterial cannula was connected to a pressure transducer (DPT100, Utah Medical Products Inc., Midvale, UT, USA). Pulsatile arterial pressure was recorded using an amplifier (Quad Bridge Amp, ML224, ADInstruments, NSW, Australia) and an acquisition board (PowerLab 4/30, ML866/P, ADInstruments, NSW, Australia) connected to a personal computer. Mean arterial pressure (MAP), systolic arterial pressure (SAP), diastolic arterial pressure (DAP), and heart rate (HR) values were derived from pulsatile arterial pressure recordings.

### Infusion of vasoactive agents

Intravenous infusion of the selective α_1_-adrenoceptor agonist phenylephrine (70 µg/ml at 0.4 ml/min/kg) and the nitric oxide donor sodium nitroprusside (100 µg/ml at 0.8 ml/min/kg) was performed using an infusion pump (K.D. Scientific, Holliston, MA, USA) [14], [25]. Phenylephrine caused incremental pressor effect while sodium nitroprusside evoked incremental depressor responses. Infusions of vasoactive drugs were randomized and the second treatment was not begun before cardiovascular parameters returned to control values. Because the cardiovascular effects of vasoactive drugs are short-lasting, with arterial pressure and HR usually returning to baseline levels within the first min after completion of infusion, interval between infusions was approximately 5 min. Infusions lasted for 20–30 s, resulting in the injection of a total dose of 9–14 µg/kg of phenylephrine and 26–40 µg/kg of sodium nitroprusside.

### Method of baroreflex evaluation

Baroreflex curves were constructed matching MAP variations evoked by intravenous infusion of phenylephrine and sodium nitroprusside with reflex HR responses. Paired values of MAP and HR were plotted to generate sigmoid logistic functions for each rat, which were used to determine baroreflex activity [25], [26]. Baroreflex analysis using sigmoid curves were characterized by 5 parameters: (i) lower HR plateau (P_1_, bpm) (i.e., maximum reflex bradycardia); (ii) upper HR plateau (P_2_, bpm) (i.e., maximum reflex tachycardia); (iii) HR range (bpm) (i.e., difference between upper and lower plateau levels); (iv) median blood pressure (BP_50_, mmHg), which is the MAP at 50% of the HR range; and (v) average gain (G, bpm/mmHg), which is the average slope of the curves between +1 and −1 standard derivations from BP_50_ [25], [26].

To analyze bradycardiac and tachycardiac responses separately, HR values matching 10, 20, 30 and 40 mmHg changes of MAP were calculated. Values were plotted to create linear regression curves for each rat and their slopes were compared to test changes in baroreflex gain [14], [27].

### Vascular reactivity to vasoactive agents

The graded changes in MAP (Δ MAP) evoked by intravenous infusion of the pressor agent phenylephrine and the depressor agent sodium nitroprusside were plotted to generate dose–response MAP curves [14], [27]. Dose–effect curves were generated for each vasoactive agent by calculating the amount of drug infused and the MAP change each 2 s after starting the infusion. The maximal effect (E_max_) and the dose at 50% of the MAP range (ED_50_) for each vasoactive agent were compared in all experimental groups.

### Cardiac morphological analysis

Formalin fixed hearts (48 h in 10% formalin) embedded in paraffin were sectioned (5 µm thick) and stained with hematoxylin and eosin (H&E). The thickness of the wall of the left and right ventricles was quantified using a video camera connected to a standard optical microscope. The left and right ventricular wall was measured using a computer-assisted image analysis system (AxioVision version 4.8.2; Carl Zeiss, Germany). Four equally spaced measurements of the left and right ventricular wall were collected from each slice, and their values were averaged. For quantification of the spaces between cardiomyocytes on left ventricle sections, five representative fields were captured using a digital camera on an optical microscope under 200×magnification. A 500 µm^2^ grid with 10×5 squares was constructed using an image editing software (Adobe Photoshop CS5, San Jose, CA, USA) and overlaid on the digital images obtained from the histological sections. The region of interest for the analysis was represented by the whole grid, which was positioned in the center of each field. The cardiomyocytes and negative area on each intersection point of the grid were recorded for quantitative assessment using a point-counting technique [28]. The results were expressed as % area of tissue (cardiomyocytes) and % negative area (space between cardiomyocytes).

Collagen interstitial fraction and cross sectional area of blood vessels on left ventricle sections were determined in Masson trichromic-stained sections. Histological images were obtained using a digital camera on an optical microscope under 200×magnification, and interstitial collagen and capillary fractional area were determined using a computer-assisted image analysis system (Image-Pro Plus, Media Cybernetic, Silver Spring, Maryland, USA).

### Drugs

Cocaine hydrochloride (Sigma, St Louis, MO, USA), phenylephrine hydrochloride (Sigma), sodium nitroprusside dihydrate (Sigma), and tribromoethanol (Sigma) were dissolved in saline (0.9% NaCl). Testosterone propionate (PharmaNostra, Rio de Janeiro, RJ, Brazil) was dissolved in almond oil. Flunixine meglumine (Banamine, Schering-Plough, Cotia, SP, Brazil) and the poly-antibiotic preparation (Pentabiotico, Fort Dodge, Campinas, SP, Brazil) were used as provided.

### Data Analysis

Data were expressed as mean ± SEM. The results were compared using one-way ANOVA followed by Bonferroni *post-hoc* test. Nonlinear regression analysis was used to compare MAP changes caused by vasoactive drugs. Results of statistical tests with *P*<0.05 were considered significant.

## Results

Neither administration of testosterone and cocaine alone nor combined for 10 consecutive days affected body weight (F_(3,20)_ = 3, *P*>0.05) (Table 1).

**Table 1:** Body and heart (absolute and relative) weight in adolescent animals subjected to treatment with testosterone and cocaine alone and in combination for 10 consecutive days.

			Heart	
Group		Body weight (g)	Absolute weight (mg)	Relative weight^a^
veh + veh	n = 5	159±6	794±86	5.0±0.6
test + veh	n = 5	171±4	738±24	4.3±0.1
veh + coc	n = 5	160±1	784±33	4.9±0.2
test + coc	n = 6	168±2	782±21	4.7±0.2

Values are mean ± SEM.

a Weight of the organ (mg)/body weight (g).

veh – vehicle, test - testosterone and coc - cocaine.

### Effect of administration of testosterone and cocaine alone or in combination on basal levels of arterial pressure and heart rate

**Figure 1:**
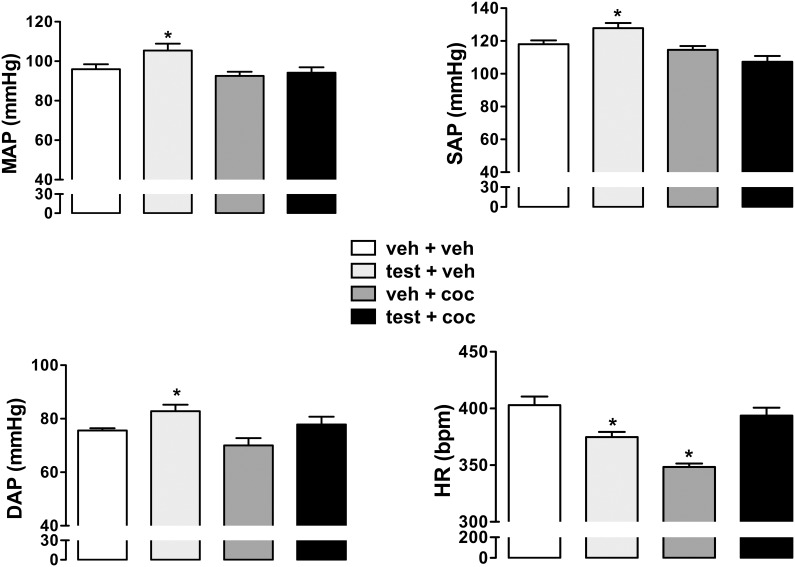
Mean arterial pressure (MAP), systolic arterial pressure (SAP), diastolic arterial pressure (DAP) and heart rate (HR) in adolescent animals subjected to treatment for 10 consecutive days with testosterone and cocaine alone and in combination. The bars represent the mean ± SEM. veh – vehicle, test - testosterone and coc - cocaine. *P<0.05 vs veh+veh group, one-way ANOVA followed by Bonferroni’s *post hoc* test.

Treatment with testosterone alone for 10 consecutive days (testosterone+vehicle group) increased basal values of both MAP (F_(3,20)_ = 4.2, *P*<0.02), DAP (F_(3,20)_ = 4.5, *P*<0.01), and SAP (F_(3,20)_ = 8.4, *P*<0.001) (Fig. 1). However, neither treatment with cocaine alone (vehicle+cocaine group) (MAP: *P*>0.05; DAP: *P*>0.05; SAP: *P*>0.05) nor in combination with testosterone (testosterone+cocaine group) (MAP: *P*>0.05; DAP: *P*>0.05; SAP: *P*>0.05) affected the basal values of arterial pressure (Fig. 1).

Treatment with testosterone+vehicle (*P*<0.05) and vehicle+cocaine (*P*<0.05) decreased basal values of HR (F_(3,20)_ = 16, *P*<0.0001), when compared with vehicle+vehicle group (Fig. 1). However, basal values of HR in animals subjected to combined treatment with testosterone and cocaine were not different those observed in control animals (vehicle+vehicle group) (*P*>0.05) (Fig. 1).

### Effect of administration of testosterone and cocaine alone or in combination in baroreflex activity

**Figure 2:**
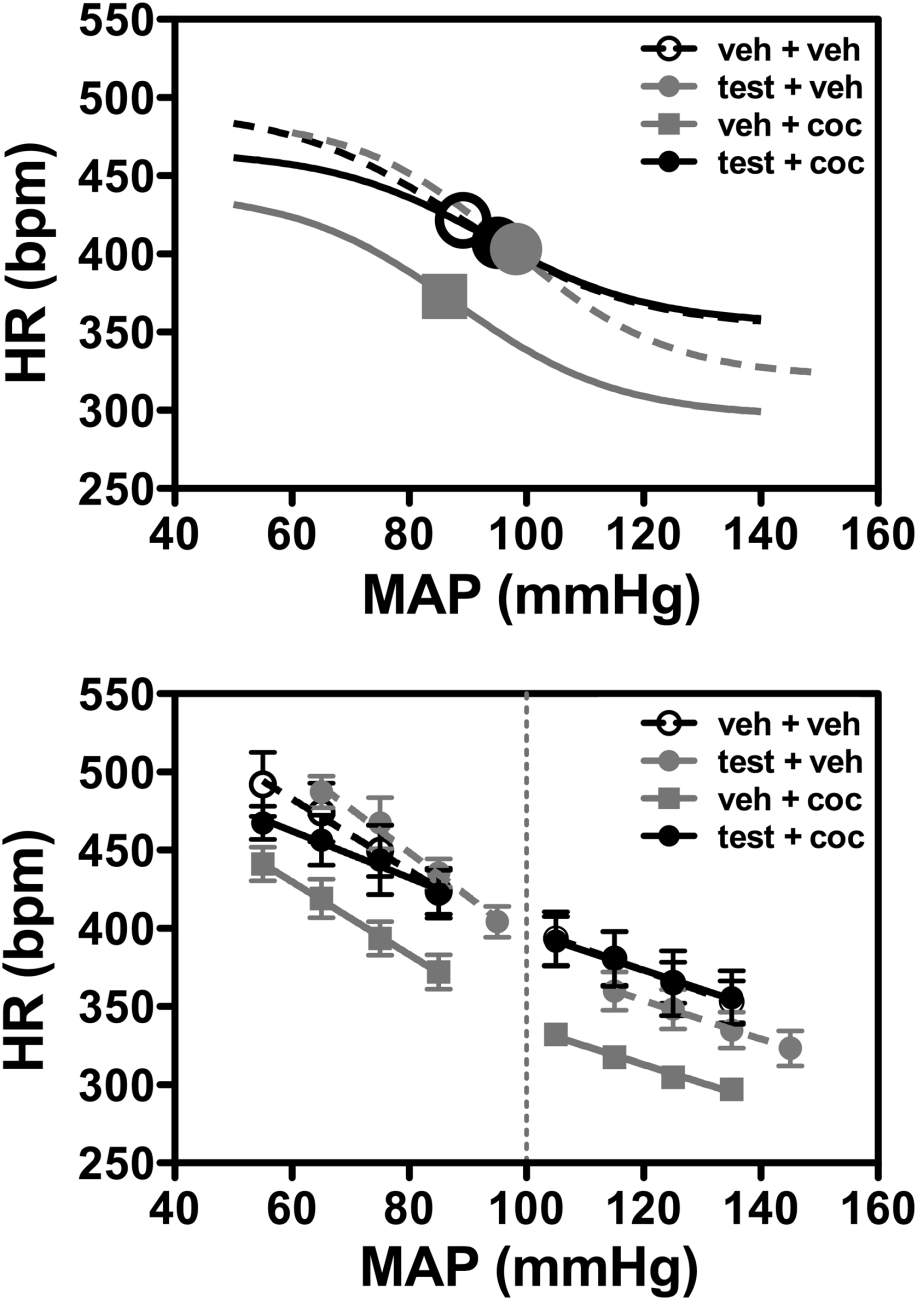
Nonlinear (top) and linear (bottom) regression analysis of the baroreflex correlating mean arterial pressure (MAP) and heart rate (HR) values in adolescent animals subjected to treatment for 10 consecutive days with testosterone and cocaine alone and in combination. Symbols on sigmoid curves (top) indicate the respective BP_50_. Circles represent the mean and bars the SEM on linear regression curves (bottom). veh – vehicle, test - testosterone and coc – cocaine. Increase or decrease in the MAP was induced by intravenous infusion of phenylephrine and sodium nitroprusside, respectively.

Nonlinear regression analysis of baroreflex activity indicated that treatment with testosterone and cocaine alone and in combination affected all parameters, except the average gain (G) (Fig. 2, Table 2). Treatment with testosterone alone (testosterone+vehicle group) decreased the maximum reflex bradycardia (lower plateau, P_1_ parameter) (P<0.01) and increased the HR range (P<0.05) and the median blood pressure (BP_50_) (P<0.05) (Fig. 2, Table 2). Daily administration of cocaine alone decreased both the maximum reflex bradycardia (lower plateau, P_1_ parameter) (P<0.001) and tachycardia (upper plateau, P_2_ parameter) (P<0.001) (Fig. 2, Table 2). Combined administration of testosterone and cocaine for 10 days decreased the maximum reflex tachycardia (upper plateau, P_2_ parameter) (P<0.05), which, in turn, decreased the HR range (P<0.01) (Fig. 2, Table 2).

**Table 2:** Parameters derived from nonlinear (G, P_1_, P_2_, HR range and BP_50_) and linear (slope bradycardia and slope tachycardia) regression analysis of the baroreflex in adolescent animals subjected to treatment with testosterone and cocaine alone and in combination for 10 consecutive days.

Group	G (bpm/mmHg)	P_1_ (bpm)	P_2_ (bpm)	HR range (bpm)	BP_50_ (mmHg)	Slope bradycardia (bpm/mmHg)	Slope tachycardia (bpm/mmHg)
veh + veh	−1.6±0.1	353±5	493±8	139±5	88±0.9	−1.4±0.3	−2.2±0.2
test + veh	−1.8±0.1	323±5*	488±5	164±6*	95±0.6*	−1.3±0.2	−2.9±0.2*
veh + coc	−1.9±0.1	297±1*	442±5*	144±4	87±1	−1.2±0.1	−2.3±0.2
test + cocaine	−1.5±0.1	356±5	468±5*	115±4*	93±2	−1.3±0.3	−1.5±0.2*
	*F(3,20) = 2*	*F(3,20) = 39*	*F(3,20) = 15*	*F(3,20) = 16*	*F(3,20) = 6*	*F(3,20) = 0.1*	*F(3,20) = 9*
	*P>0.05*	*P<0.001*	*P<0.001*	*P<0.001*	*P<0.05*	*P>0.05*	*P<0.001*

Values are mean ± SEM.

*P<0.05 vs veh+veh group. One-way ANOVA followed by Bonferroni *post hoc* test.

veh – vehicle, test - testosterone and coc - cocaine.

To further analyze the baroreflex response, the effect of either MAP increase or decrease on the HR was analyzed separately using linear regression analysis (Fig. 2, Table 2). Treatment with testosterone+vehicle for 10 consecutive days increased reflex tachycardia to blood pressure decrease (P<0.05), while combined administration of testosterone and cocaine attenuated this response (P<0.05) (Fig. 2, Table 2). Treatments did not affect bradycardiac response to blood pressure increase (*P*>0.05) (Fig. 2, Table 2).

### Effect of administration of testosterone and cocaine alone or in combination on vascular reactivity to vasoactive agents

**Figure 3:**
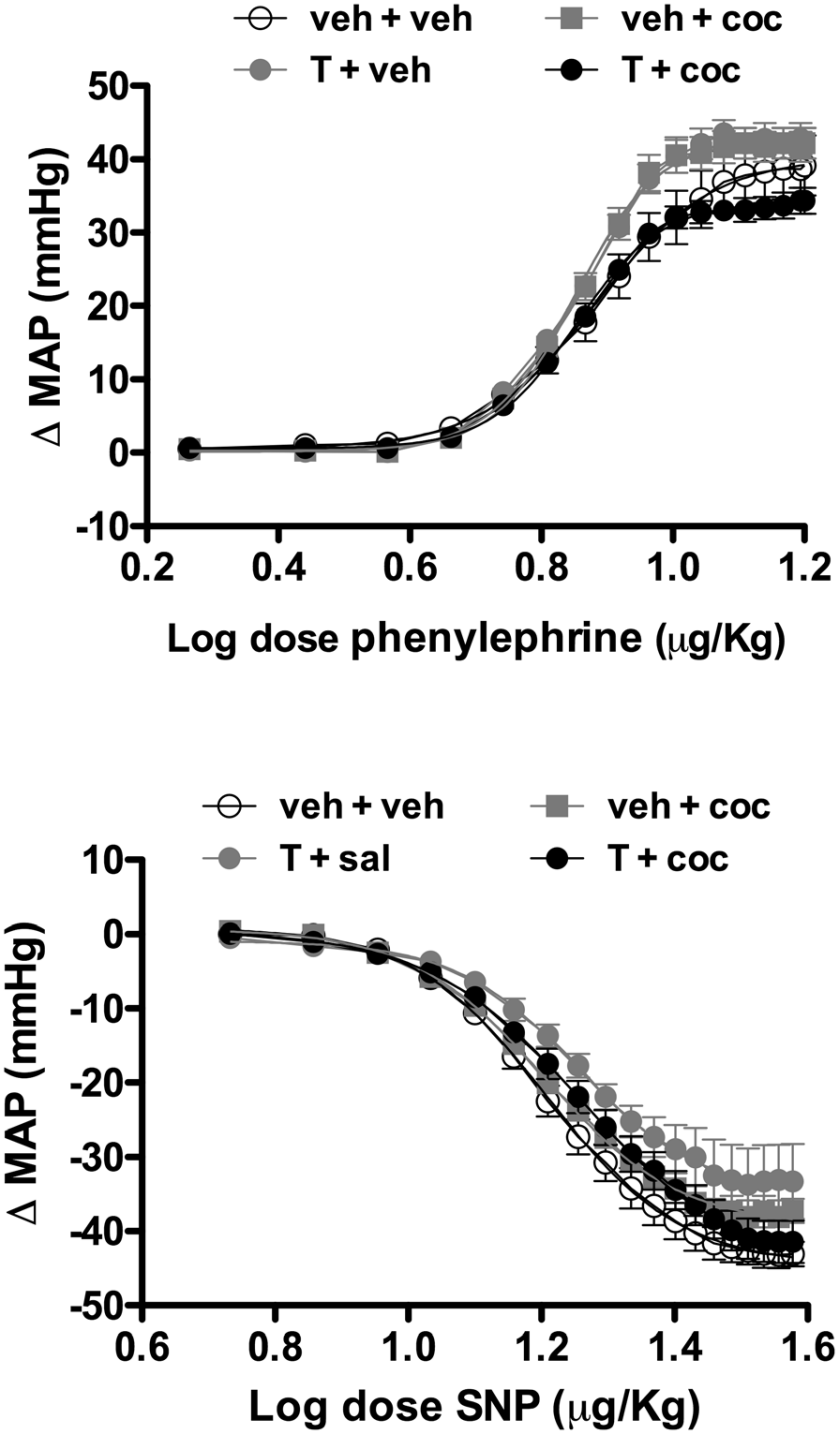
Changes in mean arterial pressure (ΔMAP) evoked by increasing concentrations of the selective α_1_-adrenoceptor agonist phenylephrine (top) and the nitric oxide donor sodium nitroprusside (SNP) (bottom) in adolescent animals subjected to treatment for 10 consecutive days with testosterone and cocaine alone and in combination. Circles represent the mean and bars the SEM. veh – vehicle, test - testosterone and coc – cocaine.

Intravenous infusion of the selective α_1_-adrenoceptor agonist phenylephrine dose-dependently induced pressor responses in all experimental groups (Fig. 3, Table 3). However, statistical analyses revealed that neither treatment with testosterone and cocaine alone nor combined affected the magnitude of the pressor response induced by phenylephrine (E_max_: *P*>0.05; ED_50_: *P*>0.05) (Fig. 3, Table 3).

**Table 3:** Maximal effect (E_max_) and dose at 50% of the MAP range (ED_50_) for phenylephrine (Phenyl) and sodium nitroprusside (SNP) dose-response curves in adolescent animals subjected to treatment with testosterone and cocaine alone and in combination for 10 consecutive days.

	Phenyl		SNP	
Group	ED_50_	E_max_	ED_50_	E_max_
veh + veh	0.86±0.02	39±4	1.34±0.03	−43±2
test + veh	0.84±0.01	42±2	1.36±0.04	−33±5
veh + coc	0.85±0.02	42±2	1.33±0.03	−37±2
test + coc	0.84±0.01	35±1	1.40±0.03	−42±3
	*F(3,20) = 0.8*	*F(3,20) = 2*	*F(3,20) = 1.5*	*F(3,20) = 2*
	*P>0.05*	*P>0.05*	*P>0.05*	*P>0.05*

Values are mean ± SEM.

One-way ANOVA followed by Bonferroni *post hoc* test.

veh – vehicle, test - testosterone and coc - cocaine.

Intravenous infusion of the nitric oxide donor sodium nitroprusside dose-dependently induced depressor responses in all experimental groups (Fig. 3). However, statistical analysis did not reveal effect of treatments with testosterone and/or cocaine in the magnitude of sodium nitroprusside-evoked decrease in arterial pressure (E_max_: *P*>0.05; ED_50_: *P*>0.05) (Fig. 3, Table 3).

### Effect of administration of testosterone and cocaine alone or in combination on cardiac morphology

**Figure 4:**
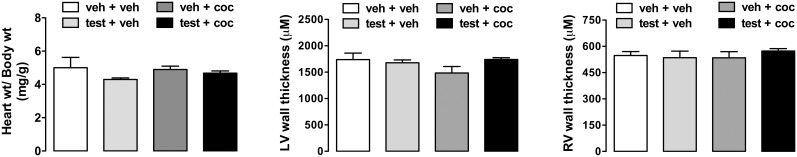
Relative weight of heart (heart weight/body weight) and left and right ventricular wall thickness in adolescent animals subjected to treatment for 10 consecutive days with testosterone and cocaine alone and in combination. The bars represent the mean ± SEM. veh – vehicle, test - testosterone and coc - cocaine. One-way ANOVA followed by Bonferroni’s *post hoc* test.

Treatments with testosterone and cocaine did not affect both absolute (F_(3,20)_ = 0.2, *P*>0.05) and relative (organ weight/body weight) (F_(3,20)_ = 1, *P*>0.05) heart weights (Fig. 4, Table 1). In addition, pharmacological treatments did not change left (F_(3,20)_ = 1, *P*>0.05) and right (F_(3,20)_ = 0.5, *P*>0.05) ventricular wall thickness (Fig. 4).

**Figure 5:**
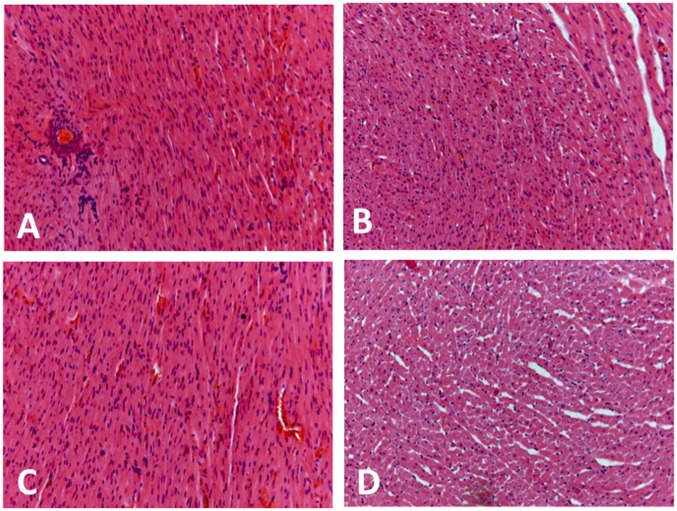
Representative H&E-stained histological sections (magnification of x200) of left ventricle from rats subjected to treatment for 10 consecutive days with testosterone and cocaine alone and in combination. (**a**) control group (vehicle+saline) showing normal cardiac muscle fibers; (**b**) testosterone+saline group and (**c**) vehicle+cocaine group showing more spaced fibers; and (**d**) testosterone+cocaine group showing similar alterations to those observed after single treatment with the substances, but changes were more pronounced.

**Figure 6:**
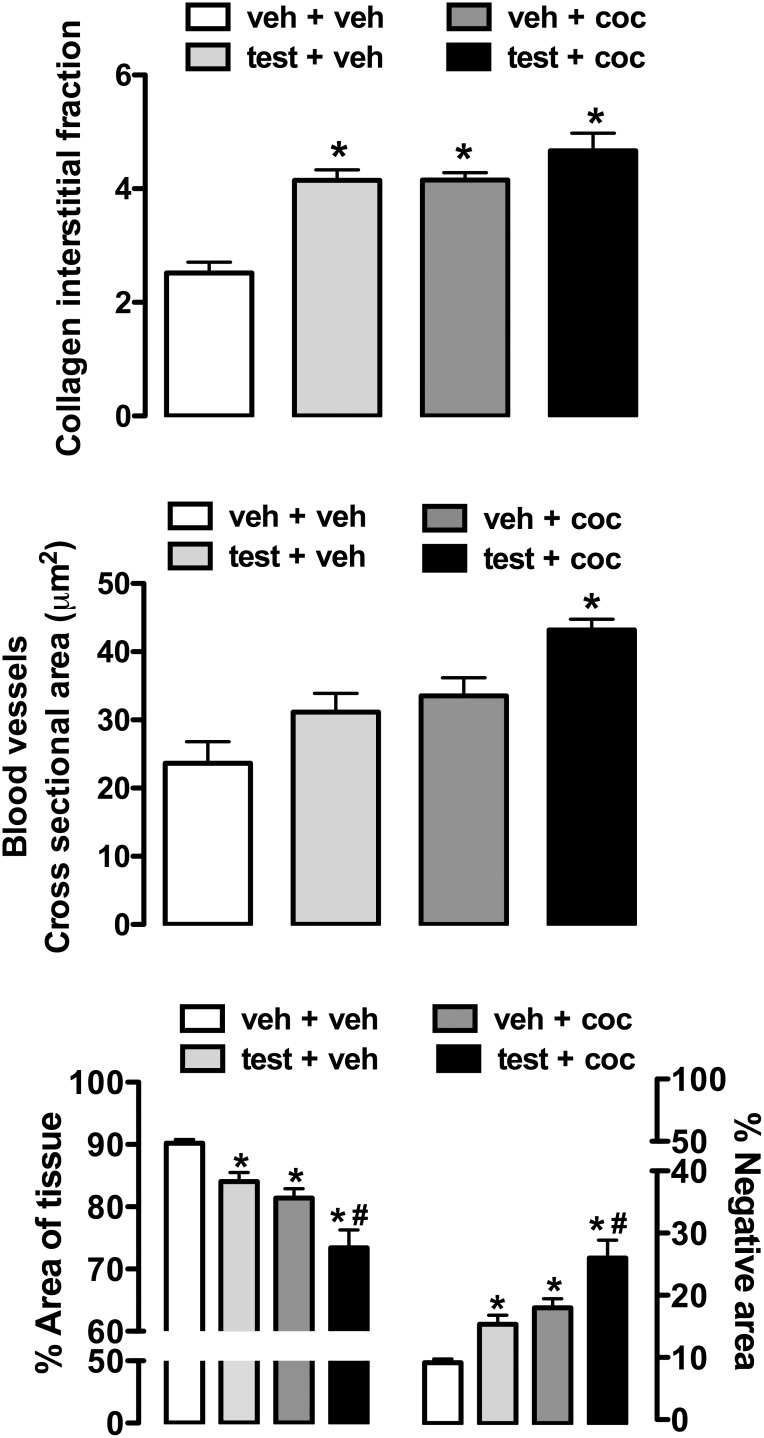
Morphometric measurements obtained in histological sections of left ventricle from adolescent rats subjected to treatment for 10 consecutive days with testosterone and cocaine alone and in combination. Collagen interstitial fraction (%) (top), cross sectional area of blood vessels (middle), and cardiomyocytes (area of tissue) and negative area (%) (bottom).The bars represent the mean ± SEM. veh – vehicle, test - testosterone and coc - cocaine. *P<0.05 vs veh+veh group, ^#^ *P*<0.05 vs test+veh and/or veh+coc groups; one-way ANOVA followed by Bonferroni’s *post hoc* test.

Figure 5a–d shows representative H&E-stained histological sections of left ventricle from rats subjected to treatment with testosterone and cocaine alone and in combination. Morphometric analysis revealed that treatment with testosterone and cocaine alone (testosterone: *P*<0.05; cocaine: *P*<0.01) and combined (*P*<0.001) increased the space between muscle fibers (F_(3,20)_ = 15, *P*<0.0001), which, in turn, reduced the area of cardiomyocytes (F_(3,20)_ = 15, *P*<0.0001). Effects were more pronounced following combined treatment with the substances (*P*<0.01) (Fig. 5 and Fig. 6).

Treatment with testosterone and cocaine alone (testosterone: *P*<0.001; cocaine: *P*<0.001) and in combination (*P*<0.001) also increased collagen interstitial fraction on left ventricle sections (F_(3,20)_ = 20, *P*<0.0001) (Fig. 6). Furthermore, combined treatment with testosterone and cocaine (*P*<0.001), but not administration of substances alone (*P*>0.05), increased cross sectional area of blood vessels on sections (Fig. 6).

## Discussion

Several pieces of evidence suggest a greater vulnerability of adolescents to the behavioral and neurochemical consequences of cocaine and AAS exposure [29]–[33]. However, the consequences of chronic exposure to these substances during adolescence on cardiovascular function are poorly understood. In the present study we report that: (i) treatment for 10 days with testosterone alone increased basal values of arterial pressure, but this effect was abolished when cocaine was coadministrated; (ii) treatment with either testosterone or cocaine alone evoked resting bradycardia, but combined treatment with these substances did not affect HR; (iii) treatment with testosterone alone increased reflex tachycardia to blood pressure decreases and reduced maximum reflex bradycardia to increase in blood pressure, while cocaine reduced both maximum reflex bradycardia and tachycardia. Combined administration of testosterone and cocaine decreased reflex tachycardia without affecting bradycardiac baroreflex response; (iv) treatments did not affect vascular reactivity to vasoactive agents; and (v) treatments did not change heart weight and ventricular wall thickness, but histological analysis of left ventricle sections showed increase in the space between muscle fibers, dilation of local blood vessels, and increase in interstitial collagen.

The arterial pressure and HR changes observed in testosterone-treated adolescent animals corroborate previous studies demonstrating that, in adulthood, chronic AAS exposure evokes sustained increases in arterial pressure and resting bradycardia [24], [34], [35]. The arterial pressure elevation is very relevant for cardiovascular function once it has been demonstrated that long-term elevations of as little as 5 mmHg in arterial pressure are associated with an increased risk of stroke and coronary heart diseases [36]. The present findings contrast with the increase in HR observed in adult rats treated for six weeks with nandrolone decanoate [37]. However, HR was measured in animals under anesthesia and effect was evidenced only after the third week of treatment [37]. A recent study investigating the consequences in adulthood of AAS exposure during adolescence did not identify significant differences in HR values of AAS-treated animals [38], thus providing evidence that cardiac chronotropic changes observed in the present study can be reversed after discontinuation of the treatment.

The absence of changes in arterial pressure in cocaine-treated rats is consistent with previous clinical and experimental reports [14], [39], [40]. However, HR reduction following cocaine exposure during adolescence contrasts with previous results from our laboratory showing absence of changes in HR in adult animals repeatedly treated with cocaine [14], [20]. The mechanisms responsible for the resting bradycardia following exposure to cocaine are not entirely understood, but may involve impaired cardiac response to sympathetic tone due to desensitization of cardiac β-adrenoceptor, changes in cytosolic and mithocondrial calcium in cardiac cells, and local generation of reactive oxygen species [40]–[42]. Furthermore, since both cocaine and testosterone treatment affect neural activity of several central nervous system structures involved in autonomic control [43]–[46], we cannot exclude a possible role of neural mechanisms in HR changes evoked by exposure to these substances.

Unexpectedly, changes in arterial pressure and HR observed after treatment with testosterone and cocaine alone were abolished when the substances were coadministrated. The absence of arterial pressure changes following combined treatment with testosterone and cocaine contrast with previous results in adult animals [19], including studies from our group [14], [24]. Furthermore, these findings are not in agreement with preclinical evidence that AAS and cocaine are capable of mutually potentiating the cardiovascular effects of each other [17], [18].

Previous studies in adult animals have reported that treatment with testosterone and cocaine alone or combined affected both the bradycardiac baroreflex response and reflex tachycardia [14], [24], [34]. However, linear regression analysis of baroreflex activity did not identify an impact of the treatments on reflex bradycardia in the present study. Therefore, reduction of lower plateau (P_1_ parameter) on sigmoid curves in testosterone- and cocaine-treated animals is mainly due to the resting bradycardia. Furthermore, changes in tachycardiac baroreflex response following treatment with testosterone alone (facilitation) or in combination with cocaine (reduction) were opposite to those observed in adulthood [14]. Linear regression analysis also did not identify differences in slope of the reflex tachycardia curve following treatment with cocaine alone, thus indicating that all alterations identified on sigmoid curves of cocaine-treated animals were due to resting bradycardia. Adolescence is a period of continuous development of the brain circuitries involved in autonomic control [47], which may explain, at least in part, the differences in baroreflex changes following treatment with testosterone and/or cocaine in adult and adolescent animals. Nevertheless, further experiments are necessary to clarify the mechanism(s) associated with discrepancies between present findings and previous reports in adult animals.

It has been proposed that impairment of baroreflex activity contributes to the development of hypertension [48], [49]. However, the elevation of arterial pressure in testosterone-treated animals was followed by a facilitation of baroreflex responses. Exacerbated tachycardiac response may be a risk factor for myocardial ischemia, sudden death and cardiac failure [50], [51]. Furthermore, despite that animals subjected to combined treatment with testosterone and cocaine did not show arterial pressure and HR changes, the reduced baroreflex activity may contribute to cardiovascular morbidity and mortality via a sympathetic overacivity as well as a reduction in cardiac parasympathetic control [52].

We have recently reported that treatment with testosterone and/or cocaine in adulthood affected vascular reactivity to vasoactive agents [14]. However, in the present study, none of the treatments affected arterial pressure responses to either the vasoconstrictor agent phenylephrine or the vasodilator substance sodium nitroprusside in adolescent rats. Therefore, although hypertension is frequently followed by vascular dysfunctions [53], the present results suggest that testosterone-induced arterial pressure increase in adolescent seems to be independent of changes in vascular function.

It has been shown that long-term exposure to testosterone and cocaine has important somatic and morphological consequences. For instance, we have previously showed that testosterone treatment during adulthood increased heart weight [14], which is in line with the well-described cardiac hypertrophic effect of the AAS [15], [16], [54]. In the same study, we also observed that cocaine treatment (alone or combined with testosterone) reduced body weight [14], corroborating other studies in adult animals [19]. However, the present data did not show significant effects of the treatments on heart weight and heart to body ratio, indicating absence of cardiac hypertrophic changes in adolescent animals. This observation was reinforced by the absence of changes on ventricular wall thickness. However, histological analysis of left ventricle sections evidenced histopathological changes after treatment with testosterone and/or cocaine, which were mainly characterized by a greater spacing between cardiac muscle fibers, dilated blood vessels, and increase in interstitial collagen. Cocaine and AAS exposure has been associated with myocarditis and myocardial oedema [10], [55], [56]. Therefore, myocardial oedema may explain the increased interstitial space observed in the present study, and myocarditis may have mediated the vasodilatation in the myocardial bed. The increase in interstitial collagen corroborates previous evidence that AAS- and cocaine-evoked myocardial injury is also characterized by myocyte necrosis and apoptosis, the presence of contraction band necrosis, and interstitial fibrosis [10], [11], [54].

Taken together with our previous findings in adult animals [14], [24], present results suggest a resilience of adolescents to the cardiovascular effects of cocaine, as well as to the cardiovascular consequences of the combined administration of testosterone and cocaine. Furthermore, adolescence seems to be an ontogenic period of resilience to effects of these substances on vascular reactivity to vasoactive agents and somatic parameters. The basis for this protection is not known. However, possible explanations include: 1) mechanisms responsible for cardiovascular changes following treatment with these substances in adulthood are protected during adolescence or not fully developed at this ontogenic period; and/or 2) control of cardiovascular function in adolescent animals is mediated by different mechanisms than adults. Alternatively, but to a smaller extent, age-related pharmacokinetic differences may also contribute to the differences observed between adolescent and adult rats [29].

In summary, our findings demonstrate that treatment with testosterone alone increases arterial pressure and evokes resting bradycardia in adolescent rats, which were independent of changes in baroreflex activity and vascular reactivity to vasoactive agents. Exacerbated reflex tachycardia observed in testosterone-treated animals may be a risk factor for cardiovascular events and sudden death. Cocaine-treated animals presented reduced baseline HR, and this effect was not related to changes in baroreflex activity. Animals subjected to combined treatment with testosterone and cocaine did not present changes in baseline arterial pressure and HR, but the reflex tachycardia was reduced. Treatments did not affect heart weight and heart ventricles wall thickness. However, histopathological changes on left ventricle sections were identified after treatment with testosterone and cocaine, and these effects were more pronounced when the drugs were coadministrated.

The present findings provide advances in our understanding of the effects on cardiovascular function of long-term exposure to cocaine and AAS alone and in combination. Nevertheless, passive injections of the substances do not necessarily mimic abuse effects. Therefore, further studies using animal models that better mimic abuse are necessary to fully assess the cardiovascular toxicity related to the abuse of cocaine and AAS.
